# Artificial Intelligence-Empowered Radiology—Current Status and Critical Review

**DOI:** 10.3390/diagnostics15030282

**Published:** 2025-01-24

**Authors:** Rafał Obuchowicz, Julia Lasek, Marek Wodziński, Adam Piórkowski, Michał Strzelecki, Karolina Nurzynska

**Affiliations:** 1 Department of Diagnostic Imaging, Jagiellonian University Medical College, 30-663 Krakow, Poland; gechrzas@cyf-kr.edu.pl; 2 Faculty of Geology, Geophysics and Environmental Protection, AGH University of Krakow, 30-059 Krakow, Poland; 3 Department of Measurement and Electronics, AGH University of Krakow, 30-059 Krakow, Poland; wodzinski@agh.edu.pl; 4 Department of Biocybernetics and Biomedical Engineering, AGH University of Krakow, 30-059 Krakow, Poland; 5 Institute of Electronics, Lodz University of Technology, 93-590 Lodz, Poland; michal.strzelecki@p.lodz.pl; 6 Department of Algorithmics and Software, Silesian University of Technology, 44-100 Gliwice, Poland; karolina.nurzynska@polsl.pl

**Keywords:** radiology, artificial intelligence, regulatory systems, AI applications, job threat

## Abstract

Humanity stands at a pivotal moment of technological revolution, with artificial intelligence (AI) reshaping fields traditionally reliant on human cognitive abilities. This transition, driven by advancements in artificial neural networks, has transformed data processing and evaluation, creating opportunities for addressing complex and time-consuming tasks with AI solutions. Convolutional networks (CNNs) and the adoption of GPU technology have already revolutionized image recognition by enhancing computational efficiency and accuracy. In radiology, AI applications are particularly valuable for tasks involving pattern detection and classification; for example, AI tools have enhanced diagnostic accuracy and efficiency in detecting abnormalities across imaging modalities through automated feature extraction. Our analysis reveals that neuroimaging and chest imaging, as well as CT and MRI modalities, are the primary focus areas for AI products, reflecting their high clinical demand and complexity. AI tools are also used to target high-prevalence diseases, such as lung cancer, stroke, and breast cancer, underscoring AI’s alignment with impactful diagnostic needs. The regulatory landscape is a critical factor in AI product development, with the majority of products certified under the Medical Device Directive (MDD) and Medical Device Regulation (MDR) in Class IIa or Class I categories, indicating compliance with moderate-risk standards. A rapid increase in AI product development from 2017 to 2020, peaking in 2020 and followed by recent stabilization and saturation, was identified. In this work, the authors review the advancements in AI-based imaging applications, underscoring AI’s transformative potential for enhanced diagnostic support and focusing on the critical role of CNNs, regulatory challenges, and potential threats to human labor in the field of diagnostic imaging.

## 1. Introduction

Humanity currently stands at the threshold of a technological revolution, in which the application of decision-making systems based on microprocessors represents a transformation on par with the invention of the wheel or the harnessing of fire [1,2,3]. The ability to transfer decision-making processes concerning the interpretation and outcomes of human-related data—traditionally reserved for the human brain—to artificial neural network systems marks a profound shift [4,5,6], representing a significant breakthrough in data processing and evaluation methods [7].

Decision-making in semiconductor-based neural networks is, to some extent, determined by humans as the problem-solving approach relies on patterns within the training data [8,9]. It is worth noting that deeper, multilayered networks have the capacity to develop autonomous decision pathways, gradually constructed based on their own iterative experiences through exposure to input data [10,11]; this process can be opaque, often resulting in solutions that surpass human intuition, as illustrated in the notable example of AI algorithms outperforming humans in the game of Go [12]. Given these capabilities, AI not only offers solutions to repetitive and time-intensive tasks but also presents novel approaches to long-standing problems, a potential that explains AI’s extensive applications in medicine [13].

AI applications are apparent in fields such as materials biology, biochemistry, and genetics where multidimensional data analysis leads to breakthroughs in creating unique structures and compounds valuable for biomaterials and pharmaceuticals [14]. AI’s utility is particularly evident in diagnostic imaging where physicians engage in the detection and classification of patterns; these tasks are time-consuming for specialized medical professionals and, due to the repetitive nature of pattern recognition, are prime candidates for AI-powered decision systems [15,16]. Consequently, developing such systems has become a priority within the scientific community, as reflected in the increasing number of algorithms aimed at detecting various anomalies in medical images [17,18]; these algorithms primarily rely on convolutional neural network architectures trained on specific image patterns [19].

The vast diversity in data encountered in modern imaging diagnostics presents substantial challenges. Algorithms must be tailored to specific tissues, organs, and imaging modalities, such as computed tomography, magnetic resonance imaging, mammography, and X-ray; this diversity is reflected in the wide array of algorithms designed to analyze various regions of the human body [20,21]. Depending on an algorithm’s validated effectiveness and integration capabilities with radiological systems, it may or may not receive endorsements from certification bodies [22,23].

In this review, the authors present an overview of existing algorithms and outline the most critical techniques used in the classification of medical imaging data, subdivided in this article into the following sections: historical perspective, deep learning solution overview, human vs. machine interaction consideration, job risk overview, and current medical solutions review.

We aim to provide a comprehensive analysis of the advancements and challenges associated with AI applications in radiology, specifically examining the evolution of convolutional neural networks (CNNs) as foundational tools in diagnostic imaging, evaluating the regulatory frameworks influencing AI integration into clinical workflows, and exploring the potential impacts of these technologies on radiologists’ roles and healthcare delivery. By synthesizing the current state of AI in radiology in this work, we seek to bridge existing knowledge gaps, propose directions for future research, and offer actionable insights for stakeholders in the medical imaging field.

This manuscript is organized as follows: In Section 2, we provide a historical background, highlighting key milestones in AI development and their impacts on medical imaging. In Section 3, we delve into contemporary deep learning models, discussing their architecture, applications, and limitations. In Section 4, we explore the comparative efficiency of AI and human radiologists, shedding light on their complementary roles in diagnostic workflows. In Section 5, we address concerns regarding the potential displacement of radiologists and emphasize the importance of balanced human–AI collaboration. In Section 6, we examine the critical role of data preparation in enhancing AI model reliability; then, in Section 7, we introduce the significance of textural analysis in medical imaging. In Section 8 and Section 9, we discuss emerging trends, challenges, and the current market landscape for AI in radiology. Finally, we conclude the manuscript with a summary of our findings and recommendations for future research.

## 2. Historical Background

The success of AI in image recognition is primarily driven by advancements in convolutional neural networks (CNNs), traced back to the application of GPU technology and modifications to the dropout technique, first implemented in 2012, which helped secure a win in the ImageNet contest. The ImageNet contest was conceived as a benchmark for evaluating the most effective approaches to image recognition, initially comprising over 10 million images across more than 22,000 categories. The breakthrough, in 2012, by Geoffrey Hinton’s students, Ilya Sutskever and Alex Krizhevsky, marked a pivotal moment in computer vision and image recognition when these researchers, initially working in speech recognition, achieved significantly better results than other competitors using two NVIDIA GTX 580 GPUs (NVIDIA Corporation, Santa Clara, CA, USA), each with 3 GB memory, to train a model with 60 million parameters over 90 epochs, completing the process in two days and presenting significantly reduced error rates in comparison to their competitors [24,25].

The success of the above approach catalyzed the broad adoption of GPUs in deep learning, a shift that represented a turning point in computer vision, in which GPUs demonstrated their efficiency in increasing the effectiveness of computer-driven image recognition. The next significant advancement, inspired by AlexNet, was ResNet, which won the 2015 ImageNet competition [26,27]; ResNet’s incorporation of residual connections (i.e., calculating output–input differences) enhanced network performance, solidifying CNNs as fundamental architecture in AI-driven image analysis [28]. The developments that followed highlighted the capabilities of CNNs and the power of GPU technology [29,30,31]. Building on these achievements, the first successful radiology-directed applications emerged, such as deep learning for lung nodule detection in the 2016 LUNA (Lung Nodule Analysis) challenge [32], which served as an early benchmark for the performance of deep learning algorithms in identifying lung nodules, thereby demonstrating the potential of AI in radiology. The introduction of deep learning models, inspired by AlexNet and encouraged in such competitions, paved the way for subsequent programs that rely on object detection within medical imaging, showcasing AI’s potential in advancing radiological diagnostics and opening new, transformative possibilities for efficient image analysis in radiology, enabling the recognition of features in radiographs, CT scans, and MRI images; these breakthroughs enabled automated feature extraction and, if broadly used, may substantially improve the accuracy of image analysis in diagnostic imaging. This progress has led to the development of AI tools that could support radiologists by providing second opinions, identifying anomalies, and even predicting disease [33].

## 3. Deep Learning Models: A Short Introduction to Current Solutions

**Figure 1:**
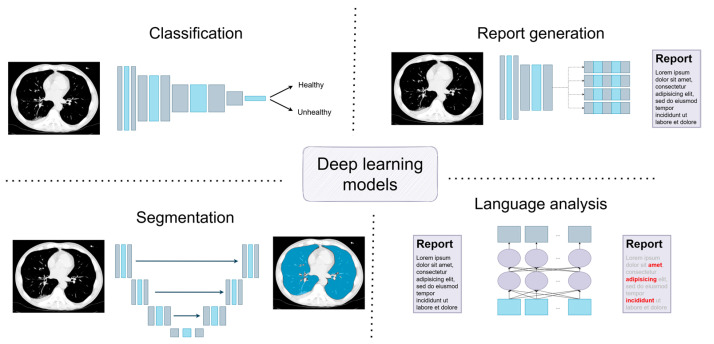
Deep learning models applicable for radiological data processing.

In radiology, several data analysis aspects should be considered, and some current deep learning solutions are depicted in Figure 1. First, a visual inspection of the myriad imagery data generated with equipment allows for insight into the human body structure. Here, the deep learning models are applied to solve the classification task between healthy and unhealthy patients, sometimes considering, more precisely, the severity stages of an illness; on the other hand, such general information might need to be more comprehensive as the determination of the region of changes, or simply segmenting the data for further analysis, is expected, in which case, convolutional neural networks are considered. However, the problem is so complex in several cases that training only one model is insufficient, and a pipeline of various models is prepared to achieve the goal.

### 3.1. Classification

When visually analyzing radiographs (X-ray), computed tomography (CT), magnetic resonance imaging (MRI), or ultrasonography scans, convolutional neural networks (CNNs) are considered. ResNet [34] is one such CNN that has gained a lot of popularity; for instance, the smallest version, ResNet-18, used for histopathological images, CT/MRI scans, and genomic data, supports prognosis in clear-cell renal cell carcinoma [35], while the largest version, ResNet-152, was applied to detect pneumonia in chest X-rays [36] and constituted part of a model dedicated to diagnosing and predicting outcomes of COVID-19 pneumonia [37]. A modified ResNet architecture was exploited for attenuation correction in pelvic PET/MR images, significantly reducing voxel-based errors and improving the quantification of bone lesions compared to other existing methods [38]; meanwhile, the 3D version of this architecture helps predict the imaging characteristics, malignancy, and pathological subtypes of pulmonary nodules detected in CT scans [39]. Of course, other architectures also find application; for example, Albiol et al. [40] compared the outcomes of ResNet-50 [34], DenseNet-121 [41], Inception_v3 [42], and Inception-Resnet_v2 [43] against radiologists’ interpretations of chest radiographs for the early detection of COVID-19, proving that the deep learning approach overcomes the experts, reaching an AUC of 0.85, compared with 0.71 in the reference group. Additionally, Fink et al. [44] showed that using Xception architecture for musculoskeletal radiographs through projection and body-side resulted in an accuracy of 0.97 in classification tasks.

Not all tasks demand large and pre-trained models; some models achieve better results with small designs but dedicated architectures. Arbabshirani et al. [45] used tailored network architecture to detect intracranial hemorrhage in head CT scans. Nguyen [46] deployed a model for accurately measuring 12 spinal alignment parameters from X-ray images, addressing the manual challenges of spinal misalignment assessment. Solak et al. [47] designed capsule networks, comprising capsules grouping neurons that collectively represent a visual entity, transforming the visual information into more representative features (e.g., height, width) and employing it to classify adrenal lesions in MR images with 0.98 accuracy.

### 3.2. Segmentation

Beyond the classification of a whole image, it is also possible to determine the detailed region where an organ or tissue of interest is depicted within an image; such a problem in computer science is called semantic segmentation, in which a mask is generated for the whole image, and each pixel is classified as belonging to some data class, consequently delineating specific regions. The encoder–decoder architecture in the U-Net structure is the most widely used for this task; for example, Gasulla et al. [48] used this approach to create a system to assess lung condition severity during the COVID-19 pandemic. Many other approaches concentrate on the segmentation of abdominal organs or the determination of body composition, in the context of muscle and fat distribution, as mentioned by Santhanam et al. [49] in their review. However, such solutions significantly impact medical imaging analysis as there are still imperfections in AI solutions when compared to human ones, as reported by Willemink et al. [50] who also claimed that applying transformer networks [51] should bring about improvements.

Although deep models have proven to be of high quality, more than one model is required in complex tasks as one usually cannot work well to meet several constraints simultaneously; therefore, in such cases, the entire process combines applying various models to achieve a goal. Larson et al. [52] designed a system composed of FastRCNN, based on ResNet-101, for landmark detection, followed by an EfficientNet-D0 model trained to recognize the presence or absence of hardware within extracted joint image patches; this system showed 99% correlation with radiologist measurements of leg length. Nurzynska et al. [53] created a pipeline, composed of two Inception_v3 networks, to determine whether the data were positive or negative for acid-fast (AF) mycobacteria; the first network removed the background, while the second network created a heatmap corresponding to the probability of AF presence. A sequence of three models was applied by Haji Maghsoudi [54] to discriminate breast cancer, in which the first model was responsible for removing the background, the second for finding and removing the pectoralis muscle—with both models based on U-Net [55] architecture—while the last one segmented dense tissue and classified it with the ResNet encoder.

As one can see, a combination of models is necessary when the determination of the main objectives requires the need to learn a system of changes or analyses; thus, usually, the first stages are responsible for removing unnecessary information, allowing the following models to concentrate more effectively on the main task.

Although the model architecture proves the solution’s success, it is not the only possibility, nor always the most optimal solution. The training algorithm chosen, especially when labeled data are absent or insufficient [53], as well as the optimization algorithm used while training the network, may influence the final outcome [56].

### 3.3. Report Generation

The application of large language models (LLM) has recently found its place in the automation of radiologist imagery description analysis. Additionally, AI technology is mature enough, and the amount of gathered data is sufficient to prepare models that automatically generate radiological image descriptions, but this does not place radiologists out of work as AI systems cannot provide a diagnosis. AI should, however, facilitate radiologists’ work with automatically generated image descriptions that they should verify and improve in detail; such an approach may improve early diagnosis and limit the radiologist’s workload [57]. For example, Zhang et al. [58] developed a generative model to automate the generation of radiological reports from chest X-rays. Blind assessments by radiologists indicated that the generated reports were comparable in quality to those produced by human experts. Another approach was presented by PRAS Bassi et al. [59] who introduced RadGPT, an anatomy-aware vision-language AI agent designed to generate detailed reports from CT scans. RadGPT segments tumors, including benign cysts and malignant growths, along with surrounding anatomical structures, transforming this information into both structured and narrative reports. These reports provide comprehensive details on tumor size, shape, location, attenuation, volume, and interactions with adjacent blood vessels and organs.

Conversely, Sun et al. [60] evaluated GPT-4’s ability to generate the “Impressions” section of radiology reports from the “Findings” section, comparing its outputs to those of human radiologists. Radiologists rated human-generated impressions higher in coherence, comprehensiveness, and factual consistency and concluded that, despite GPT-4’s potential to assist in report generation, its outputs currently do not match the quality of human radiologists’ work.

### 3.4. Language Analysis

The functionality of LLMs in radiology is not restricted to generating reports but includes broader applications across the field. For instance, LLMs have shown potential in accurately identifying and classifying true and false laterality errors in radiology reports [61]. Enhancing patient understanding of their condition is crucial for improved outcomes, making the creation of more patient-friendly imaging reports an essential area for LLM implementation. As Butler et al. [62] demonstrated in the context of orthopedic radiology, AI-LLM may improve the readability of radiological reports across multiple imaging modalities. Moreover, LLMs can serve as effective tools for post hoc structured reporting in radiology, enabling significant time savings by automating the organization and structuring of radiological data for improved efficiency and accessibility [63]. LLMs have also been employed to classify unstructured radiology reports into standardized categories. For example, a feasibility study by Matute-González et al. developed LiverAI, a specialized large language model designed to automatically annotate free-text MRI reports with LI-RADS v2018 categories. The findings revealed that incorporating LiverAI into clinical processes could streamline workflows by reducing the radiologists’ workload by 45% while maintaining high diagnostic accuracy [64].

These advancements underscore the versatility of LLMs in radiology, highlighting their capacity to improve diagnostic accuracy, patient communication, and workflow efficiency across various applications.

## 4. Are Machines More Efficient than Human Doctors?

Radiology involves the interpretation and classification of medical images based on characteristic features; a radiologist is trained to distinguish between features considered pathological and normal, identifying abnormalities and grouping them under specific diagnostic categories [65,66]. The process of extracting common features from sets of known images represents a combination of knowledge and expertise, along with the innate pattern recognition skills of the imaging specialist. This ability to recognize shared features across random datasets can be described mathematically and is often viewed as a measure of cognitive intelligence as the capability to achieve specific goals based on these generalizations is a classic marker of system intelligence [67,68]. Machine learning has shown promise in replacing aspects of this process as machines can emulate intelligence by generalizing and identifying similarities across varied models with limited connections because the ability to find similarities across patterns is understood as a background of artificial intelligence [69]. Furthermore, human knowledge dissemination is inherently slow, limited by communication bottlenecks—dictation or writing proceeds at only a few bits per second, even with templates, which remains considerably slower than machine processing [70]. A significant challenge in radiology is the diversity in data, which vary not only among imaging modalities (e.g., CT, MRI, CR) but also within the same anatomical structures, which can convey differing information, thus requiring a deep understanding of imaging processes and extensive training [71]. Additionally, the vast range of pathologies is challenging to encode in numerical formats that are suitable for machine processing; however, recent advances show that machines with self-learning capabilities can efficiently extract and replicate patterns, demonstrating an impressive ability to recognize features and share learned patterns swiftly [72,73]—progress which has motivated various research groups to create specialized programs designed to extract specific pathological features. However, there is no comprehensive program that can accurately detect all pathological features in any given image (i.e., a generalized AI for pathology detection); currently, radiology relies on a suite of AI tools, each tailored to address a particular problem. Familiarity with these tools is essential for radiologists to work effectively, especially as the gap between available radiology professionals and the volume of medical images continues to grow. The widespread use of AI tools in radiology could enhance diagnostic accuracy, reduce error rates, and improve patient outcomes by standardizing processes and expediting diagnoses.

**Figure 2:**
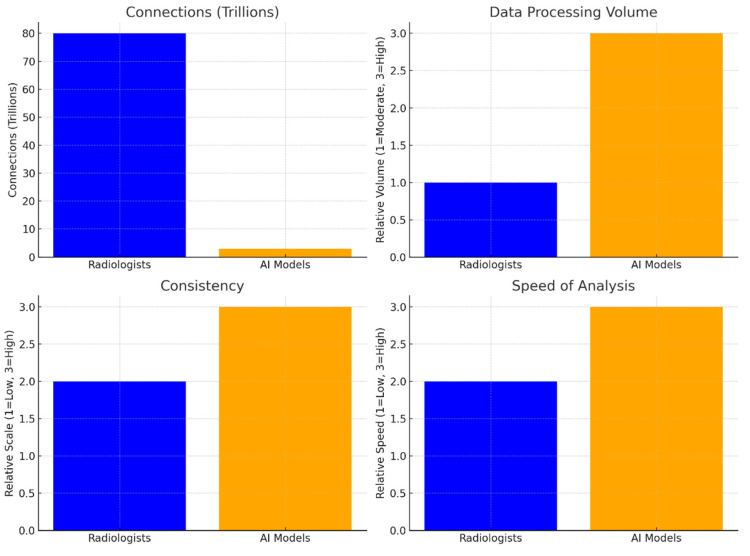
Detailed comparison of human radiologists and AI models, emphasizing the unique strengths each brings to medical imaging tasks.

Starting with connections, radiologists, represented by the human brain, have approximately 80 trillion synaptic links, highlighting their complex neural network that enables deep adaptability and perceptual sensitivity. In contrast, AI models, with around 3 trillion parameters, have fewer connections, which points to their efficiency with structured data but reveals potential limitations in adaptability (Figure 2). Moving to data processing volume, radiologists handle a moderate amount of data in each session, reflecting their focus on quality over quantity and their ability to adapt to diverse cases [74].

However, AI models surpass radiologists in their ability to process large volumes of data quickly, which is advantageous for tasks requiring speed and consistency, but AI models may lack the nuanced adaptability seen in human analysis. When it comes to consistency, radiologists show moderate reliability, as their work is often subject to slight variability due to factors such as fatigue and cognitive biases; AI models, on the other hand, demonstrate high consistency, performing uniformly across datasets and maintaining precision without the effects of fatigue or bias [75].

The high consistency of AI models makes them suitable for tasks that require repetitive accuracy though they may not capture subtleties as effectively as humans. In terms of speed of analysis, radiologists typically exhibit a moderate pace, balancing thoroughness with the need for careful evaluation; their work involves a nuanced approach that can be time-consuming but essential for accuracy in complex cases. AI models, in contrast, excel in speed, rapidly processing images in high volumes, which proves useful for scenarios in which rapid diagnostics are critical though it often requires human validation for complex findings [76].

The above comparison highlights the complementary nature of radiologists and AI models: radiologists bring adaptability, perceptual depth, and expertise, while AI models contribute efficiency, speed, and consistent accuracy. Together, these strengths suggest that an integrated approach, combining human insight with AI efficiency, might offer the most comprehensive and effective path forward in medical imaging diagnostics.

## 5. Is the Job of a Radiologist at Risk?

Machines are often considered superior to humans in solving logical problems; however, in terms of perception—especially in complex medical image recognition—humans still outperform machines [77], and this remains true when we consider a radiologist’s ability to adapt to diverse datasets and detect various pathologies [78,79,80]. The following two primary factors determine the performance of both biological and artificial networks: first, the number and architecture of connections, which dictate the network’s ability to capture intricate patterns; and second, the volume and quality of data as better data typically yield better outcomes, a principle often summarized in the phrase “garbage in, garbage out” [81]. Additionally, there are other factors, unique to artificial networks, which are not directly comparable to human brain processes but significantly influence artificial network performance, including training techniques, optimization algorithms, hyperparameter tuning, model generalization, and transfer learning [82,83]. Biological systems, such as the human brain, contain an enormous number of connections—up to 80 trillion synaptic links—whereas large language models typically rely on around 3 trillion parameters [84,85,86] (Table 1), revealing a fundamental difference between the two: while humans operate with many connections and a relatively low data volume, machines possess vast data volumes but operate with relatively fewer connections. Machine learning models’ information extraction abilities are proportional to their capacity to identify similarities across datasets, including visual patterns, which can be measured with data compression tests [69,87]; on the other hand, foundation models trained on extensive datasets demonstrate high proficiency in detecting pathologies and, when well-trained, can outperform human observers in terms of accuracy, sensitivity, consistency, and speed [88], without suffering from fatigue or biases that commonly limit humans [89,90,91]. Currently, radiologists, equipped with broad and adaptable knowledge, remain the most proficient operators of relevant tools, which addresses the pressing question regarding the future of radiology [77,92,93]; the unique insights and adaptability of radiologists suggest that their expertise will remain vital in integrating AI tools into medical practice [94].

## 6. Importance of Data Preparation for Processing with AI

**Figure 3:**
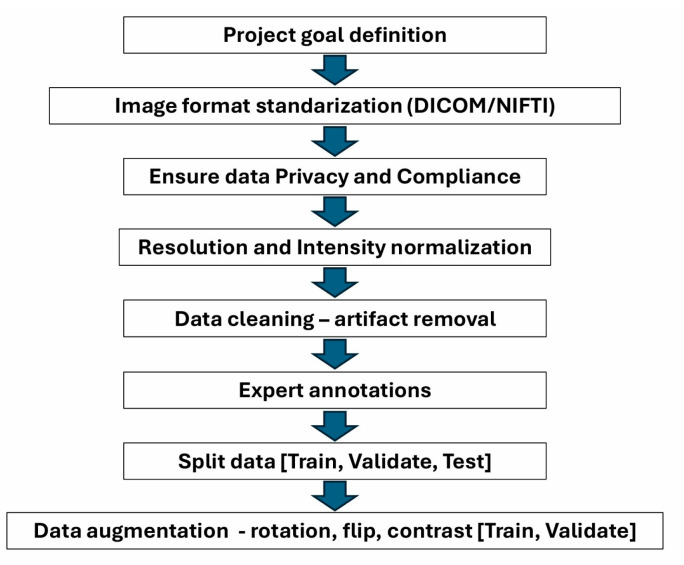
Necessary steps in medical imaging data preparation for processing with AI.

Preparing medical imaging data for machine learning (ML) requires a systematic approach to ensure model reliability, accuracy, and generalizability (Figure 3). First, clear project goals guide data preparation as classification, segmentation, and detection tasks each have unique requirements [95]. Standardization is essential, including converting images to a consistent format (e.g., DICOM or NIfTI) and normalizing resolution and intensity to account for differences across modalities such as X-ray, MRI, or CT [96]. Data cleaning addresses artifacts (e.g., motion blur) and ensures label accuracy, while quality assurance (QA) checks verify noise levels, contrast, and anatomical coverage. Accurate annotation by experienced radiologists is critical, with guidelines established to maintain consistency, especially for complex tasks such as segmentation. Data augmentation techniques (e.g., rotations, flips, contrast adjustments) expand limited datasets, increasing robustness. For data augmentation, techniques that are appropriate to the problem and logical should be used. It is crucial that data augmentation techniques align with the clinical context to avoid introducing unrealistic transformations. For example, while minor rotations (a few degrees left or right) can enhance the robustness of models, extreme rotations such as 90 degrees are inappropriate for X-ray imaging as they do not reflect how such images are typically analyzed by clinicians. After preparation, datasets should be split into training, validation, and test sets while preventing leakage by keeping patient images in only one subset. Balanced datasets are ideal, but, in smaller datasets, k-fold cross-validation can be helpful to maximize data use. Metadata management and privacy safeguards are essential, and patient information should be de-identified to comply with regulations (e.g., HIPAA or GDPR). Finally, pre-training checks, including basic statistical analysis and baseline model evaluation, help identify potential issues before training [97].

## 7. The Role of Textural Analysis in Image Preprocessing

Image texture is a crucial component of various image types, including medical images, as medical imaging modalities visualize the properties of internal organs and tissues through textural representation; for instance, the textures observed in tomographic cross-sections provide valuable diagnostic insights. Textural parameters are part of radiomics [98] and reflect the physiological characteristics of tissues, enabling applications such as organ segmentation, lesion detection, and the assessment of pathological changes. The importance of textural analysis in diagnostic imaging has been established across various modalities, including computed tomography (CT) [99], magnetic resonance imaging (MRI) [100], and ultrasound [101].

Furthermore, variations in acquisition parameters across different patient images often affect brightness and contrast in regions of interest (ROIs); these variations can occur between consecutive images, causing some textural features to depend not only on texture but also on factors such as average brightness and contrast. Consequently, features intended to describe tissue structure may inadvertently reflect scanner sensitivity inconsistencies within the analyzed region [102], potentially leading to inaccurate tissue characterization or misclassification.

In order to mitigate these issues, the normalization of ROIs is commonly applied, typically serving as an initial step and expanding the image histogram within the ROI to cover the entire available intensity range, a process that enhances the contrast between bright and dark structural elements within the texture and reduces the influence of local mean intensity, thereby improving the quality of extracted features. Different methods for ROI normalization, as well as their influence to the overall image analysis results, are presented in reference [103].

Texture feature maps also play important roles in the analysis of biomedical images. A feature map is an image of the distribution of values of a given textural parameter, determined for the entire image or its fragment; the value of a feature is determined for each image point in its neighborhood large enough to enable the correct characterization of the texture. A feature map allows us to observe how well a given feature distinguishes the analyzed textures; in the case of a properly selected parameter, a map of the feature transforms the textured areas into relatively uniform fragments differing in brightness (thereby encoding the values of the selected parameter).

**Figure 4:**
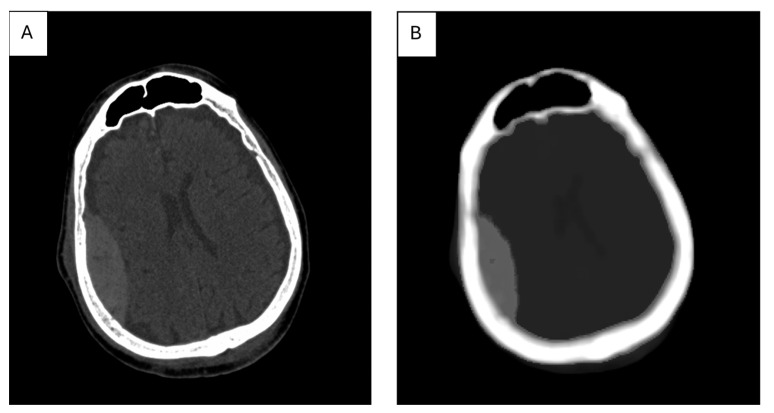
CT image of a brain cross-section with a marked stroke area (**A**); AngularSecondMoment feature map calculated for this image, in which a 15 × 15-pixel window was used (**B**).

The map—or, more often, maps—of features selected during the selection process are used as input data for the segmentation stage. An example feature map for an image of a brain cross-section, containing a fragment of a subarachnoid hemorrhage, is shown in Figure 4 where the map of the AngularSecondMoment feature (calculated based on the Gray Level Cooccurrence Matrix, GLCM) [104] contains the stroke area, which has a significantly different value than the rest of the brain; therefore, the image of this map allows us to isolate the stroke area using simple brightness thresholding.

**Figure 5:**
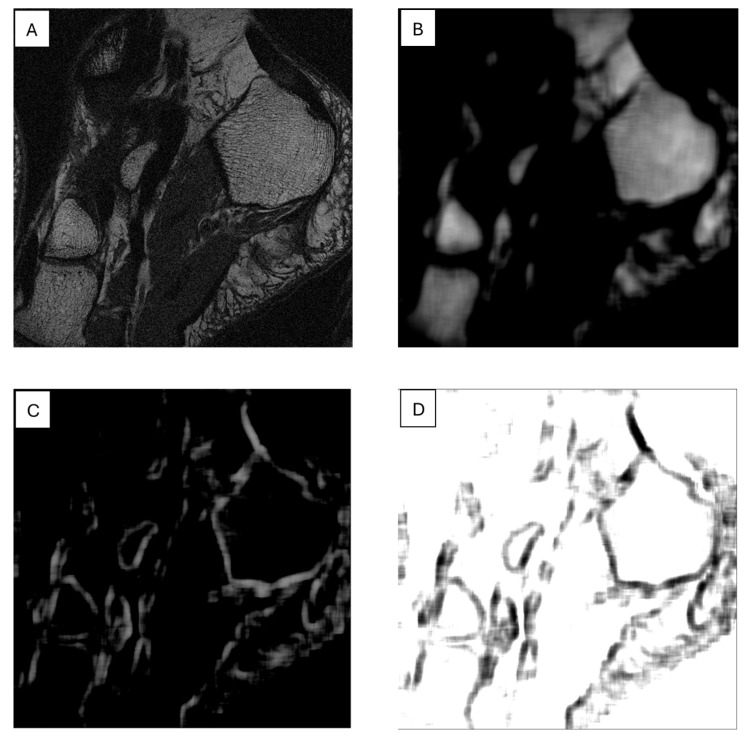
T1W MR image of the foot bone (**A**); feature maps calculated for this image: SumAverage (**B**); SumOfSquares (**C**); and Sigma (**D**).

Another example of using such maps for image segmentation is shown in Figure 5, determined for the SumAverage parameter from GLCM for a 15 × 15-pixel sliding window across the T1W MR image of a foot cross-section; bone regions are shown on this map as smooth areas with a small range of brightness levels, which allows them to be relatively easily distinguished from other regions. Texture feature maps can also be used for edge detection. Figure 5C,D shows such maps, obtained for the SumOfSquares (GLM) and Sigma (autoregressive model) features, respectively, in which the edges shown define bone tissue regions. An example of a texture map application combined with an active contour model for biomedical image segmentation is discussed in reference [105].

## 8. AI Is Supportive but Must Be Used with Caution

Although AI-based solutions have numerous applications in radiology, e.g., they represent state-of-the-art technology used for image segmentation [106,107], there are several applications for which they are suboptimal or should be used with caution.

Firstly, deep learning-based contributions to medical image registration are still less accurate than methods based on classical numerical optimization [108,109,110,111,112]. In an experiment classifying the patient sex using automated analysis of computed tomography scans of vertebrae, standard machine learning, used for the classification of textural features (classical approach), achieved an accuracy of 69%, while deep convolutional networks for this task yielded a slightly lower accuracy of 59% [113]. AI-based algorithms tend to have problems with generalizability to previously unseen cases, requiring further instance-level optimization in order to achieve optimal results; for example, in the large-scale Learn2Reg benchmark, all of the best-performing methods used classical optimization to further improve the learning-based results for both the brain MRI and abdominal CT tasks [108]. The same observations apply to the BraTS-Reg challenge [109], which was dedicated to comparing different algorithms against pre-operative and post-surgery registration of brain MRIs; all of the best-performing solutions used traditional numerical optimization and outperformed the AI-based contributions. Moreover, classical numerical optimization methods, such as PDE-constrained optimization, have been shown to be highly effective in medical image analysis, providing reliable and high-fidelity results compared to deep learning-based solutions, especially in applications requiring precise alignment, such as neuroimaging and cardiovascular imaging [114]. Therefore, while deep learning-based methods show promise, they still lag behind classical numerical optimization techniques in terms of accuracy and generalizability. Further research is needed to enhance the performance of AI-based algorithms and address their limitations [115].

Another area in which AI methods should be used with caution is image generation and reconstruction; nowadays, people attempt to use AI to generate new synthetic volumes for image augmentation [116] to transfer one modality to another (e.g., to generate synthetic CT from MR to avoid irradiation) [117], to directly create synthetic volumes using textual prompts [118], or to accelerate image reconstruction [119]. Although these applications are becoming increasingly successful, people should use these methods with special care because they often tend to generate non-existing structures that may misguide medical experts [120]. Deep learning models used for MR-to-CT synthesis have demonstrated impressive results, but careful validation is necessary in order to ensure clinical reliability [121]; reconstructed images should be compared with standard sequences to ensure no degradation or unintended alternations in image quality and anatomy have occurred [122].

AI-based contributions are often incorrectly evaluated [123], leading to incorrect conclusions; for example, most of the contributions in automatic image segmentation evaluate accuracy using annotations from just a single annotator rather than reporting confidence intervals [124,125]. However, if the ground truth is based on annotations from human experts, then any evaluation should be always performed using ground truths annotated by several radiologists and inter-variability should be evaluated. Moreover, deep learning methods inherently suffer from problems with standardization and explainability [126], making it difficult to compare results across different studies and ensure consistency in performance metrics [127]. Even though there are significant attempts to improve the interpretability and explainability of deep learning methods, we are still far from clear interpretations of their performed decisions and recommendations [128].

Lastly, one should remember the vulnerability of CNN-based architectures to adversarial attacks, a significant concern in the field of deep learning, as they involve making small, often imperceptible, changes to input images that can cause a CNN to make incorrect predictions. Even a single pixel modification or added learned adversarial pattern to the input may completely fool a deep network [129], a situation that may be dangerous because external modifications to the input volumes, completely invisible to radiologists, may completely change the neural network recommendations. Even though there are methods with which to detect adversarial attacks using feature response maps, the users of CNNs should be aware of the risk [130]; more recent architectures, based on vision transformers (ViTs), are more resistant to certain types of adversarial attacks compared to CNNs [131], but it is important to note that no model is entirely immune to such abuse [132].

## 9. Review of AI Products Used in Radiology: Status in 2024

The review of AI products used in radiology in 2024 was conducted utilizing the two following key databases: the Health AI Register [133] and AI Central [134]. The Health AI Register lists AI products available in the European market, all of which are CE-marked, indicating compliance with European regulatory standards; conversely, AI Central focuses on AI tools cleared by the FDA and commercially available in the United States. Our dual-database approach ensured comprehensive coverage of the radiology AI landscape, and the review process involved counting and cataloging available products, analyzing their distribution across radiological subspecialties, imaging modalities, and targeted diseases. Regulatory certifications (CE marking and FDA clearance) were systematically reviewed, alongside data on the year of market entry and FDA clearance. This review was conducted in October 2024 to capture the most current state of the field.

**Figure 6:**
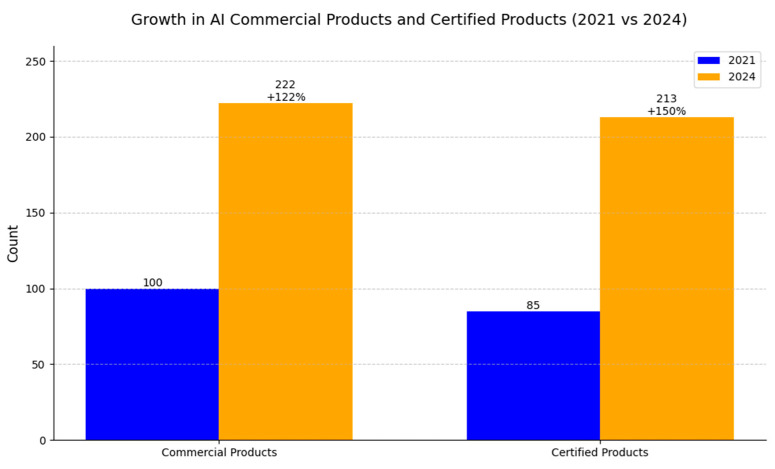
A bar graph illustrating the growth in the number of working AI algorithms and certified products between 2021 and October 2024. The annotations highlight the counts for both years and the percentage increases, emphasizing the rapid expansion of AI solutions in healthcare.

The current status of AI applications used in Europe was reviewed on the basis of the Health AI Register [133], which provides the names of applications along with their subspeciality modality and type of certification approved in the EU market. The same system was used for a similar analysis by Van Leuven in 2021 [20]; however, the landscape of the AI market has since significantly changed, and our analysis reflects the rate of development for AI solutions in the healthcare sector, revealing that, as of October 2024, there are 222 commercial AI-based products, representing an increase of 122%—among these, 213 products are reported to be certified, marking a 150% increase compared to the 85 certified products reported in 2021 (Figure 6).

**Figure 7:**
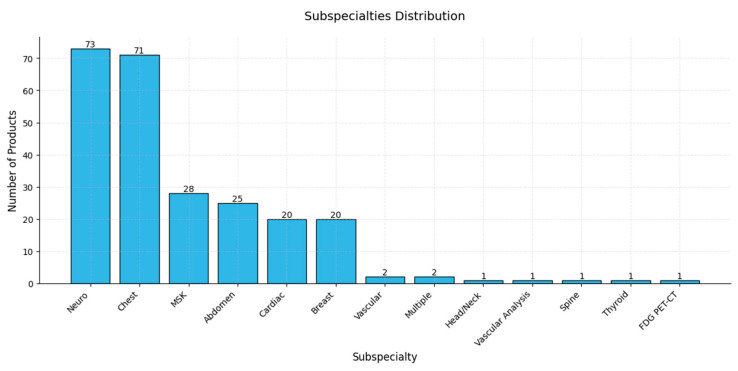
A bar chart illustrating the distribution of AI-based products across various medical subspecialties. The *x*-axis lists the different subspecialties, while the *y*-axis indicates the number of AI products available in each area.

The focus on neuroimaging and chest imaging, with 73 and 71 AI products, respectively, suggests a strong emphasis on developing AI applications in these areas, which may be attributed to high clinical demand and the complexity of interpreting neuroimaging and chest images, making these subspecialties ideal for AI innovation [135,136]. Areas such as musculoskeletal (MSK), abdominal, cardiac, and breast imaging also show considerable development, with product counts between 20 and 28, indicating significant interest and activity; in contrast, subspecialties including vascular, head/neck, spine, thyroid, and FDG PET-CT imaging have relatively few AI products (between 1 and 2), possibly reflecting lower demand, limited dataset availability, or less complex imaging challenges [137,138,139]. The above distributions highlight the concentrated development of AI tools in high-demand areas of medical imaging, with the potential for further growth as new needs and opportunities emerge across other subspecialties (Figure 7).

**Figure 8:**
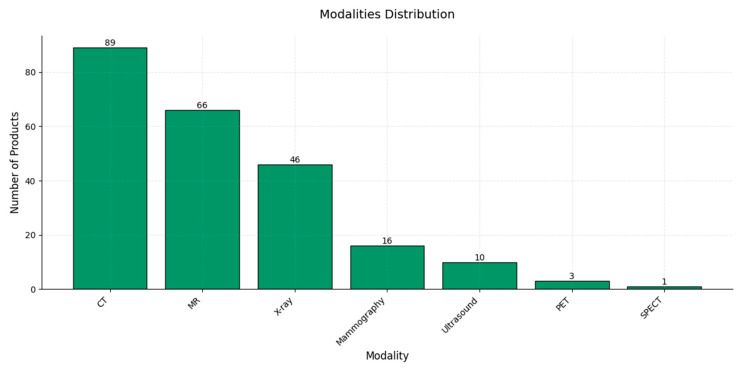
A bar chart illustrating the distribution of AI-based products across various imaging modalities. The *x*-axis represents different imaging modalities—CT, MR, X-ray, mammography, ultrasound, PET, and SPECT—while the *y*-axis shows the number of AI products available for each modality.

In terms of imaging modalities, CT and MR stand out, with the highest numbers of AI products—89 and 66, respectively—likely due to the extensive use and high diagnostic value of these modalities, as well as the complexity of their data, which makes them particularly well-suited for AI applications [140]. X-ray imaging follows, with 46 products, emphasizing a strong focus on AI in this widely utilized modality [141]; meanwhile, mammography and ultrasound, with 16 and 10 products, respectively, exhibit moderate levels of AI development, likely due to the specialized nature of mammography and the inherent variability of ultrasound imaging, which present unique challenges for AI algorithms. PET and SPECT, with only 3 and 1 products, respectively, represent the lowest levels of AI development among the imaging modalities, possibly due to their lower usage rates and specialized applications in nuclear medicine [142]. Overall, this distribution reflects the alignment of AI product development with high-demand, complex imaging modalities within the healthcare sector (Figure 8).

**Figure 9:**
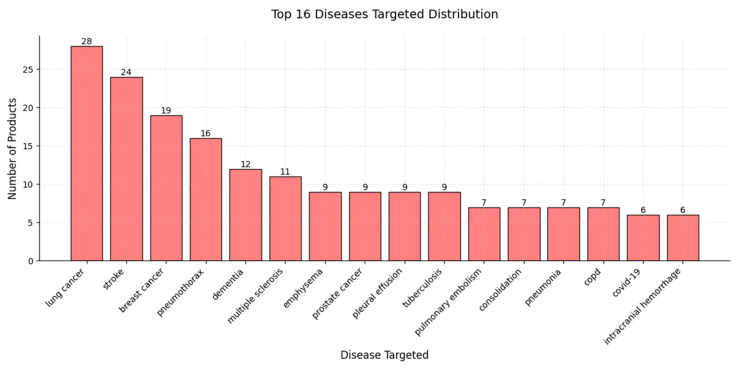
A bar chart illustrating the distribution of AI products with regard to their top 16 targeted diseases. The *x*-axis lists the targeted diseases, while the *y*-axis represents the number of AI products developed for each condition.

Regarding targeted diseases, lung cancer, stroke, and breast cancer lead, with 28, 24, and 19 AI products, respectively, underscoring the prevalence and clinical importance of these conditions [143] and suggesting that the complexity of diagnosis and the high clinical impacts of these diseases make them attractive areas for AI applications. Other notable areas of AI development include pneumothorax, with 16 products, and dementia, with 12 products, indicating growing interest in respiratory and neurodegenerative conditions. Moderate levels of AI product development are seen in diseases such as multiple sclerosis (11 products), emphysema (9 products), prostate cancer (9 products), pleural effusion (9 products), and tuberculosis (9 products), indicating valuable opportunities for AI applications in these areas as well. Diseases including pulmonary embolism and consolidation, pneumonia, COPD, COVID-19, and intracranial hemorrhage, with product counts ranging from 5 to 7, represent the least targeted conditions, potentially reflecting emerging areas in which AI applications are still in the early stages. Targeted disease distribution indicates a strong focus on high-impact, high-prevalence diseases [144,145] while also highlighting the potential for continued growth in AI-driven solutions for a broader range of diseases [146,147,148,149,150,151] (Figure 9).

**Figure 10:**
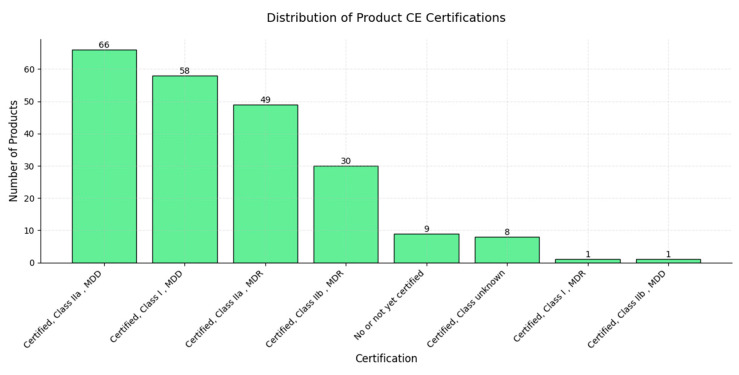
A bar chart displaying the distribution of CE certifications across various AI products. The *x*-axis represents different certification categories, while the *y*-axis shows the number of products in each category.

A majority of AI products are certified as Class IIa under the Medical Device Directive (MDD), totaling 66 products, followed by Class I under the MDD, with 58 products, and Class IIa under the Medical Device Regulation (MDR), with 49 products; these categories represent the bulk of certified AI products, indicating a high level of compliance with established regulatory standards. Class IIb under the MDR comprises 30 products, showing a significant but smaller representation, while there are 9 products that are not yet certified or have no certification, suggesting that they may be in development or awaiting regulatory approval. Smaller categories include “Certified, Class unknown” (8 products), “Certified, Class I, MDR” (1 product), and “Certified, Class IIb, MDD” (1 product). The concentration of AI products in Class IIa and I certifications under both the MDD and MDR reflects a strong effort to comply with regulatory frameworks, especially for products targeting moderate-risk classifications; this distribution underscores the critical role of regulatory certification in the development and deployment of AI products in healthcare (Figure 10).

**Figure 11:**
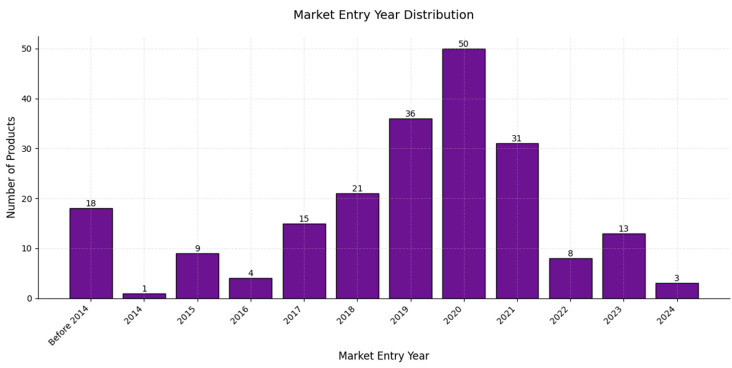
A bar chart illustrating the distribution of AI products by the market entry year. The *x*-axis represents the market entry years, ranging from “Before 2014” to 2024, while the *y*-axis shows the number of products that entered the market in each year.

In terms of the market entry year, AI product releases before 2014 represent a notable group, with 18 entries, reflecting early AI market activity; from 2014 to 2016, there was a gradual increase in market entries, though still with small numbers each year; however, beginning in 2017, a steady rise occurred, with 15 products in 2017 and 21 in 2018, an upward trend that reached a peak in 2020, with 50 products, indicating a surge in AI adoption, likely driven by technological advancements and growing market demand [152]. Although 2021 saw a slight decline, to 31 products, it still represented high market activity; from 2022 to 2024, the number of entries continued to decrease, with 8, 13, and 3 products, respectively, possibly reflecting a maturing market, saturation in product development, or a shift in focus toward other healthcare areas—a trend which demonstrates the rapid expansion and growth of the AI market in healthcare, with a potential move toward saturation in recent years [153,154,155,156,157]. The peak in AI radiology product approvals in 2020 can be attributed to several key factors: during this period, a wealth of COVID-19 imaging datasets became publicly available, offering researchers the crucial data needed to train and validate AI models effectively; additionally, significant increases in funding and grants were directed toward AI research, particularly in response to the pandemic [158] (Figure 11).

**Figure 12:**
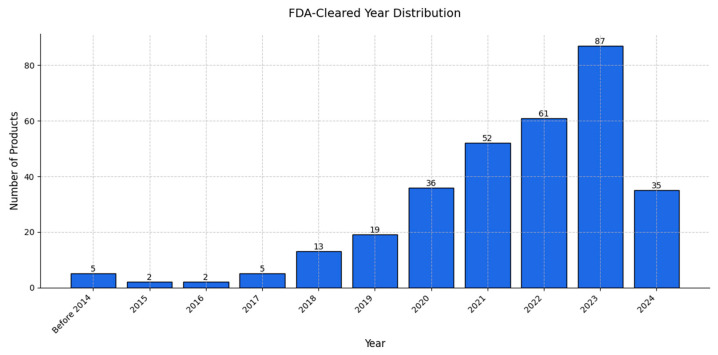
A bar chart illustrating the annual number of AI products cleared for use in radiology between 2008 and 2024. The trend reached its peak in 2023, with over 80 products cleared that year, reflecting the rapid growth and adoption of AI technologies in healthcare during this period. The year 2024 shows a slight decline, potentially signaling market stabilization or shifts in regulatory processes. The progression depicted here highlights the increasing integration of AI solutions into clinical practice, particularly in medical imaging.

The chart in Figure 11 depicts the market entry year distribution for AI products, while that in Figure 12 presents the annual number of AI products cleared for use in radiology from 2008 to 2024. A comparison of these data provides insights into the dynamics of AI adoption in radiology as we can observe a significant growth phase starting around 2017, with surges in product entries and clearances. The peak in market entries occurred in 2020, with 50 new products entering the market, while product clearances peaked slightly later, in 2023—a lag that suggests that products entering the market require time for regulatory approval [159,160].

After 2020, the number of new product entries declined steadily, with only three entries recorded in 2024; this trend reflects a possible stabilization or maturation of the AI radiology market in which fewer groundbreaking entries were being introduced. The slight decline in product clearances in 2024, following a peak in 2023, suggests market saturation. The regulatory landscape may be stabilizing and the focus might be shifting, from introducing new products to refining and deploying existing solutions [161].

The initial growth phase and subsequent stabilization observed in Figure 11 and Figure 12 highlight the natural lifecycle of an emerging technology market, in which the early years are marked by innovation and rapid expansion, leading to a peak, followed by market consolidation and saturation as competition and regulatory frameworks mature [162,163].

In this work, we emphasize the rapid development in the AI medical market in recent years. Three years ago, Van Leeuwen et al. [20] described the landscape of artificial intelligence (AI) products in radiology, identifying 100 commercially available CE-marked solutions in the European market. In the current work, on the basis of the same vendor-supplied noncommercial database, we were able to distinguish over 220 products (www.radiology.healthairegister.com (accessed on 28 August 2024)), a result that is unlikely to reflect all available products, which becomes evident when different databases are compared, for instance, AI and radiology databases (https://aiandradiology.com (accessed on 30.08.2024)), used to collect and review products present in the domestic Polish market. The Health AI Register database is the largest and most reflective as it is the most comprehensive database, including the process of certification. Three years ago, 85 out of 100 reported products were validated, while, currently, 213 out of 222 registered products to date are certified among the different classes (CE I–III), representing an important shift toward their acceptance by medical boards and easing their implementation into different medical institutions (Figure 3). Wu [164] investigated the adoption and usage of over 500 FDA-approved AI medical devices in the U.S. in 2023, focusing on 16 specific procedures that are billable through AI-specific CPT codes, spanning a variety of medical domains, including cardiology, ophthalmology, radiology, and liver health, with the most prevalent applications being coronary artery disease and diabetic retinopathy. Recently, in the U.S., 700 cleared AI algorithms were reported by Fornell, with 76% present in radiology but covering various specialties (radiology: 527; cardiology: 71; neurology: 16; hematology: 14; gastroenterology and urology: 10; clinical chemistry: 7; ophthalmic: 7; general and plastic surgery: 5; anesthesiology: 5; pathology: 4; microbiology: 4; general hospital: 3; orthopedic: 3; ear, nose, and throat: 2; and dental: 1). The above comparison illustrates the differences between the European and U.S. markets and reflects adoption obstacles within the European market, indicating a significantly larger size of the U.S. market and forecasting rapid development in the future [165,166].

Varghese [167] described several key challenges in the clinical adoption of artificial intelligence in medicine, organizing them under the RISE framework. Regulatory approval and sufficient evidence remain significant hurdles as many AI systems lack the rigorous prospective and multicenter clinical trials necessary for validation [22,168,169]; additionally, the majority of studies focus on retrospective or theoretical aspects, delaying their translation into clinical practice. Interpretability also poses a barrier as high-performing AI models, particularly those utilizing deep learning architectures, often lack transparency in their decision-making processes although clinicians are more likely to adopt AI systems that provide human-understandable explanations for their outputs. Additionally, interoperability challenges hinder the integration of AI systems into clinical workflows as effective communication between diverse hospital information systems, while preserving the meaning and context of medical data, is required. Finally, AI systems depend on high-quality and structured data, which are often limited, and reliance on unstructured sources, such as free-text medical records, introduces ambiguity and inaccuracies. The need to address these challenges through robust validation processes explains why AI adoption, despite a need for efficient automated systems in healthcare, is still limited. The development of integrating platforms and the formation of universal algorithms will, therefore, boost further AI implementation into medical practice.

## 10. Examples of Practical Implementation of AI Models

The safe, effective, and high-quality deployment of AI technologies is critical for advancing medical practice. Several medical environments provide exemplary use cases of robust AI models in detecting various pathologies and improving diagnostic workflows. In 2020, a multihospital experiment in Moscow [170] tested AI solutions for chest X-ray analysis across 178 state healthcare centers. AI frameworks analyzed redirected X-rays to detect abnormalities without prior training data. A top-performing framework employed advanced techniques such as EfficientNets and DenseNet, analyzing 17,888 cases over one month with an overall AUC of 0.77, ranging from 0.55 for herniation to 0.90 for pneumothorax. Robert et al. [171] evaluated the impact of AI as a second reader in detecting and localizing lung nodules on chest radiographs (CXRs) from 40 hospitals across the U.S. The study showed that AI assistance significantly improved diagnostic performance, with the mean AFROC increasing from 0.73 to 0.81 and AUROC from 0.77 to 0.84, along with a sensitivity improvement from 72.8% to 83.5%.

These results highlight AI’s potential as a tool to enhance diagnostic accuracy without increasing false positives. Sacha et al. [172] assessed an AI system for detecting clinically significant prostate cancer on MRI using a retrospective cohort of 10,207 MRI examinations and a multi-reader study with 62 radiologists. The AI system demonstrated superior performance compared to radiologists (AUROC: 0.91 vs. 0.86) and detected 6.8% more significant cancers at the same specificity, emphasizing its potential as a diagnostic support tool. In a meta-analysis by Wang [173], the effectiveness of deep learning algorithms for detecting and segmenting brain metastases on MRI was evaluated. The study identified 42 relevant studies and assessed them using QUADAS-2 and CLAIM tools. The results showed a pooled lesion-wise dice score of 79% and sensitivities of 86% (patient-wise) and 87% (lesion-wise). U-Net models performed best, with accuracy influenced by MRI hardware diversity and slice thickness. These findings underscore deep learning’s promise in brain metastasis diagnostics. Lu et al. [174] conducted a randomized, multi-reader, multi-case study to assess AI-assisted auto-contouring for brain tumor stereotactic radiosurgery (SRS). Nine professionals contoured brain tumors in assisted and unassisted modes. AI significantly improved inter-reader agreement (DSC from 0.86 to 0.90, *p* < 0.001) and lesion detection sensitivity (91.3% vs. 82.6%, *p* = 0.030). Additionally, AI assistance enhanced contouring accuracy and reduced time spent by 30.8%, particularly benefiting less-experienced clinicians. Salehi et al. [175] performed a meta-analysis on AI algorithms detecting primary bone tumors, comparing their diagnostic performance to clinicians. Internal validation showed AI sensitivity and specificity at 84% and 86%, respectively, compared to clinicians’ 76% and 64%. In external validation, AI achieved 84% sensitivity and 91% specificity, while clinicians reached 85% and 94%. With AI assistance, clinicians improved sensitivity to 95% but experienced reduced specificity (57%). This study highlights AI’s potential and emphasizes the need for further optimization. Bachmann et al. [176] evaluated the impact of an AI tool on nonspecialist readers detecting traumatic fractures in appendicular skeleton radiographs. Using a multi-reader, multi-case design with 340 radiographic exams, sensitivity increased from 72% to 80% and specificity from 81% to 85% with AI assistance (*p* < 0.05). Missed fractures decreased by 29%, and false positives by 21%, without affecting reading time. The greatest improvement was in detecting nonobvious fractures, demonstrating AI’s potential to enhance diagnostic performance efficiently. Jalal et al. [177] highlighted AI’s role in emergency radiology, emphasizing its ability to enhance imaging analysis, workflow efficiency, and patient care quality, and mitigate radiologist burnout. Ketola et al. [178] proposed a structured evaluation process for AI applications in radiology, including pre-evaluation, retrospective testing, and prospective clinical integration, to ensure safety, effectiveness, and compatibility with clinical standards.

## 11. Summary

Artificial intelligence has shown transformative potential in the field of radiology, revolutionizing how medical imaging is interpreted and utilized in clinical practice as AI technologies have demonstrated the ability to automate a wide range of tasks, thereby significantly enhancing efficiency and diagnostic accuracy. Tasks ranging from image segmentation, abnormality detection, and classification of imaging data to more advanced processes, such as report analysis and automated case triaging, are reshaping traditional radiological workflows, thus allowing radiologists to focus on complex cases and critical decision-making, while routine processes are efficiently handled using AI.

In recent years, the field of radiology has experienced an explosion of innovative AI applications as the research has introduced numerous methodologies that leverage deep learning, machine learning, and other AI techniques to address specific challenges in medical imaging; concurrently, the market has witnessed the emergence of many commercial AI products, some of which have obtained regulatory certifications, indicating their readiness for clinical deployment and underscoring the growing roles of AI in both research and practice.

Despite the potential of AI, it is imperative to recognize the ongoing importance of standard image analysis techniques, such as textural analysis and radiomics; these methods, which are rooted in well-established statistical and geometric principles, remain valuable tools for extracting meaningful features from medical images. The advantage of these features is their partial medical interpretability, which is not represented in deep learning models [113]; combining these standard approaches with AI techniques has the potential to create hybrid systems that leverage the strengths of both methods, thus yielding robust, interpretable, and clinically useful outcomes.

Moreover, ethical considerations and adherence to regulatory standards are critical for the successful integration of AI into radiology as protecting patient privacy and ensuring data security must remain fundamental priorities. AI developers and healthcare institutions must comply with the stringent regulations governing the use of medical data in order to maintain trust and integrity in healthcare systems. Additionally, transparency in AI development, from dataset selection to model validation, is vital for addressing potential biases and ensuring equitable outcomes.

In conclusion, while AI offers unprecedented opportunities to advance radiology, its integration requires a balanced approach; acknowledging the continued relevance of standard methods, addressing ethical and regulatory challenges, and fostering a collaborative relationship between radiologists and AI are essential for realizing its full potential. By combining the strengths of AI and traditional methodologies, radiology has the opportunity to achieve new levels of precision, efficiency, and impact in medical imaging.

The combined insights from the data reviewed in this manuscript suggest that the AI radiology market has transitioned from a phase of rapid growth to one of stabilization and potential saturation, a trend that reflects the increasing maturity of the field, in which regulatory processes and product deployment are catching up with the initial surge in innovation. Future efforts may focus more on optimizing existing solutions, rather than introducing entirely new products.

## Figures and Tables

**Table 1:** Detailed comparison of human radiologists vs. AI models [78,79,80,81,82,83,84,85,86,87].

Factor	Radiologists	AI Models
**Data Processing Volume**	Moderate	High
**Connections (Trillions)**	80	3
**Adaptability**	High	Low
**Perception of Patterns**	High	Moderate
**Consistency**	Moderate	High
**Speed of Analysis**	Moderate	High
**Fatigue Resistance**	No	Yes
**Bias Resistance**	No	Yes
**Training Techniques Required**	No	Yes

## Data Availability

Not applicable due to the absence of additional data beyond those included in the content of the work.
